# Nearly Identical Plasmids Encoding VIM-1 and Mercury Resistance in Enterobacteriaceae from North-Eastern Germany

**DOI:** 10.3390/microorganisms9071345

**Published:** 2021-06-22

**Authors:** Stefan E. Heiden, Katharina Sydow, Stephan Schaefer, Ingo Klempien, Veronika Balau, Peter Bauer, Nils-Olaf Hübner, Katharina Schaufler

**Affiliations:** 1 Pharmaceutical Microbiology, University of Greifswald, 17489 Greifswald, Germany; stefan.heiden@uni-greifswald.de (S.E.H.); katharina.sydow@uni-greifswald.de (K.S.); 2 MVZ Laboratory Limbach Vorpommern-Rügen, 18435 Stralsund, Germany; stephan.schaefer@labor-stralsund.de; 3 Klinische Hygiene und Infektiologie, Helios Hanseklinikum, 18435 Stralsund, Germany; ingo.klempien@helios-gesundheit.de; 4 IMD Laboratory Greifswald, Institute of Medical Diagnostics, 17493 Greifswald, Germany; veronika.balau@imd-greifswald.de; 5 Centogene, 18055 Rostock, Germany; peter.bauer@centogene.com; 6 Central Unit for Infection Prevention and Control, University Medicine Greifswald, 17475 Greifswald, Germany; nils.huebner@med.uni-greifswald.de

**Keywords:** VIM, Enterobacteriaceae, mercury, resistance, IncN

## Abstract

The emergence of carbapenemase-producing Enterobacteriaceae limits therapeutic options and presents a major public health problem. Resistances to carbapenems are mostly conveyed by metallo-beta-lactamases (MBL) including VIM, which are often encoded on resistance plasmids. We characterized four VIM-positive isolates that were obtained as part of a routine diagnostic screening from two laboratories in north-eastern Germany between June and August 2020. Whole-genome sequencing was performed to address (a) phylogenetic properties, (b) plasmid content, and (c) resistance gene carriage. In addition, we performed phenotypic antibiotic and mercury resistance analyses. The genomic analysis revealed three different bacterial species including *C. freundii*, *E. coli* and *K. oxytoca* with four different sequence types. All isolates were geno- and phenotypically multidrug-resistant (MDR) and the phenotypic profile was explained by the underlying resistance gene content. Three isolates of four carried nearly identical VIM-1-resistance plasmids, which in addition encoded a mercury resistance operon and showed some similarity to two publicly available plasmid sequences from sources other than the two laboratories above. Our results highlight the circulation of a nearly identical IncN-type VIM-1-resistance plasmid in different Enterobacteriaceae in north-eastern Germany.

## 1. Introduction

The increasing occurrence of resistances to carbapenems is of concern and has been frequently detected not only in clinical settings but also the community, livestock and food products [1,2,3]. Carbapenems are last-line antibiotics that are mainly used to treat infections caused by Gram-negative pathogens, where more conventional antibiotics such as cephalosporins and quinolones are no longer effective [1]. For the last decade, the prevalence of carbapenemases in Gram-negative bacterial pathogens has been increasingly described [1]. These bacteria are the cause of life-threatening infections among humans and animals, including sepsis, pneumonia and urinary tract infections. Resistance to carbapenems is mainly conferred through bacterial production of carbapenemases such as VIM, NDM and IMP. Italy reported VIM-1 (for “Verona integron-encoded metallo-beta-lactamase”) as one of the first metallo-beta-lactamases (MBL) [4]. MBLs are enzymes that hydrolyze penicillins, different cephalosporins, and carbapenems but are susceptible to aztreonam. MBL-encoding pathogens have been reported worldwide [5,6]. In Europe, most VIM-producing isolates belong to Enterobacteriaceae with *Klebsiella pneumoniae* being one of the most common [7]. Notably, MBL production is often accompanied by cross- and co-resistances resulting in multidrug-resistant (MDR) phenotypes. The enzyme-encoding genes are mostly carried on large resistance plasmids that transfer among different bacteria [1]. VIM-positive isolates are often unrelated but clonal epidemics have also been reported. Their emergence across different hospitals has been for example observed in Spain and Italy [8]. For an outbreak in Greece, several *K. pneumoniae* isolates were found to have *bla*_VIM-1_ as part of a class 1 integron that also carried additional antibiotic resistance genes. Mostly, the integron was located on transferable incompatibility (Inc) N plasmids. [9,10]. IncN plasmids are associated with a large variety of antimicrobial resistances and can be transferred via conjugation among a broad host range [11]. In addition, several other plasmid types were described to carry VIM-encoding genes such as IncF, IncA/C or IncL/M [12].

Here, we characterize four VIM-positive isolates obtained in 2020 from two laboratories in Mecklenburg-Western Pomerania, Germany and highlight the circulation of a nearly identical *bla*_VIM-1_ and mercury resistance plasmid.

## 2. Materials and Methods

Between June and August 2020, we collected four enterobacterial isolates from three different patients as part of a routine sampling program at two laboratories in Mecklenburg-Western Pomerania, Germany (MVZ Laboratory Limbach Vorpommern-Rügen, Stralsund, and IMD Laboratory Greifswald). Initial antimicrobial susceptibility testing (AST) was performed using the VITEK 2 (bioMérieux, Germany) system and the AST cards N214 and N389. Bacterial species were initially identified by MALDI-TOF MS (Bruker, Germany). An immunochromatography lateral-flow test RESIST4 by Coris BioConcept was performed to differentiate the carbapenem resistance and identify VIM-1.

All four VIM-positive isolates were whole-genome sequenced (WGS) on an Illumina NextSeq 550 (Microbial Genome Sequencing Center [MiGS], Pittsburgh, PA, USA). One isolate (*E. coli* PBIO2728) was additionally long-read sequenced at MiGS using ONT’s Nanopore system. Two isolates were obtained from the same patient. DNA was extracted using the MasterPure™ DNA Purification Kit for Blood, Version II (Lucigen, Middleton, WI, USA). After quantification and initial quality control, DNA was shipped to MiGS (Pittsburgh, PA, USA) and following library preparation sequenced using 2 × 150 bp paired-end reads. Raw sequencing reads were adapter-trimmed, contaminant-filtered and quality-trimmed using BBDuk from BBTools v. 38.86 (http://sourceforge.net/projects/bbmap/; accessed on 15 April 2021). Both raw reads and trimmed reads were quality-controlled using FastQC v. 0.11.9 (http://www.bioinformatics.babraham.ac.uk/projects/fastqc/; accessed on 15 April 2021). De novo genome assembly was conducted using shovill v. 1.1.0 (https://github.com/tseemann/shovill; accessed on 15 April 2021) in combination with SPAdes v. 3.14.1 [13]. Briefly, as part of the shovill workflow, genome size was estimated, and trimmed reads subsampled if they exceeded a coverage of 100× (+10%). The trimmed reads were then error-corrected and overlapping read pairs merged. After assembly with the de Bruijn graph assembler the draft genomes were corrected by mapping the reads back to the resulting contigs and after sorting and processing of the alignments, variants were called. Finally, contigs, which were too short, low coverage or consisting of homopolymers were removed from the assembly. We included a second polishing step after the pipeline by mapping the trimmed reads back to the contigs with BWA v. 0.7.17 [14]. The resulting SAM/BAM files were sorted and duplicates marked with SAMtools v. 1.10 [15]. Variants were then called with Pilon v. 1.23 [16]. For PBIO2728 we obtained additional long-read sequencing data and assembled the genome in a hybrid approach (short and long reads) with Unicycler v. 0.4.8 [17]. We used Prokka v. 1.14.6 [18] for automatic annotation.

The in silico multi-locus sequence typing (MLST) and antibiotic resistance gene detection were carried out using mlst v. 2.19.0 (https://github.com/tseemann/mlst; accessed on 15 April 2021) and ABRicate v. 1.0.0 (https://github.com/tseemann/abricate; accessed on 15 April 2021), respectively. Both tools rely on 3rd-party public databases (e.g., PubMLST [19], PlasmidFinder [20]). IncN plasmids were typed in silico with the three-locus plasmid MLST (pMLST) scheme [21]. Single nucleotide polymorphism (SNP) detection between PBIO2728 and a publicly available genome of E-124-4 *E. coli*, an isolate with the same sequence type (ST10) (NCBI accession NZ_PDDP00000000.1) was performed using snippy v. 4.6.0 (https://github.com/tseemann/snippy; accessed on 15 April 2021).

We visualized BLAST comparisons with BRIG v. 0.95-dev.0004 [22] and NCBI BLAST v. 2.11.0+ [23] and created a synteny plot with genoPlotR v. 0.8.9 [24]. 

Plasmid profile analysis for all isolates was performed as previously described [25]. 

As for the phenotypic mercury tolerance tests, overnight cultures of four isolates were adjusted to McFarland standard 0.5, and 100 μL of a 1:100 dilution of adjusted suspensions in Mueller-Hinton broth (Roth, Karlsruhe, Germany) was used as inoculum for incubations for 16 to 20 h at 37 °C in mercury-containing microtiter plates (Thermo Scientific Nunc plates, Schwerte, Germany) with concentrations of 500-0, 50-0 and 10-0 µg/mL Hg. After incubation, the minimal inhibitory concentration was determined visually and reported as the tolerance breakpoint. Experiments were performed with three technical and three biological replicates. *K. pneumoniae* PBIO1953 (ST307) with known mercury susceptibility was used as control isolate [26].

## 3. Results

All four isolates carried the *bla*_VIM-1_ gene and were geno- and phenotypically multidrug-resistant. Following the interpretation criteria of the European Committee on Antimicrobial Susceptibility Testing (EUCAST: Breakpoint tables for interpretation of MICs and zone diameters. Version 11.0, 2021. https://www.eucast.org; accessed on 23 May 2021), all isolates were resistant to third-generation cephalosporins, carbapenems, piperacillin-tazobactam, aminoglycosides and trimethoprim-sulfamethoxazole. PBIO2728 was intermediate resistant to ciprofloxacin.

The bacterial species included *C. freundii* (PBIO2726, *n* = 1), *E. coli* (PBIO2728, *n* = 1) and *K. oxytoca* (PBIO2729, PBIO2730, *n* = 2) (Table 1).

In addition to VIM resistance, whole-genome sequencing revealed carriage of different antimicrobial resistance genes such as *sul*, *tet* and *aad* (Table 1 and Table S1), which matched the observed resistance phenotypes (e.g., *sul/dfr*: resistance to trimethoprim-sulfamethoxazole, *aac/aad*: resistance to gentamicin, *bla*_VIM-1_: resistance to ceftazidime, carbapenems and piperacillin-tazobactam). One isolate (PBIO2730) carried *mcr-9* but showed no phenotypic resistance to colistin. Previous studies reported that this member of the *mcr* gene group does not necessarily confer phenotypic colistin resistance although overexpression in *E. coli* led to increased minimal inhibitory concentrations (MICs) [27,28]. 

Hybrid assembly of PBIO2728 with short and long read sequencing data successfully reconstructed five replicons: the chromosome (4,667,605 bp), plasmid 1 (76,002 bp; incompatibility [Inc] type: IncN ST7: *repN*(3), *traJ*(4), *korA*(2)), plasmid 2 (62,310 bp; IncFIA, IncFIB, IncFII), plasmid 3 (6647 bp), and plasmid 4 (2101 bp). Plasmid 1 carried the *bla*_VIM-1_ gene.

**Figure 1:**
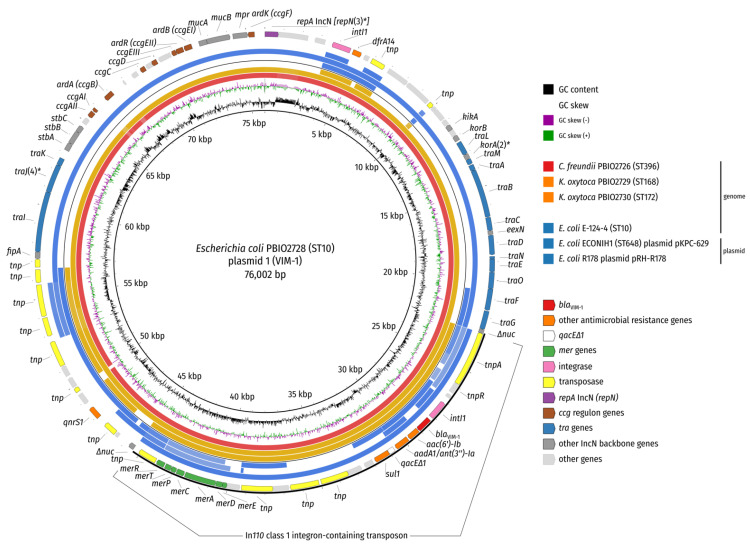
BLAST comparison of plasmid 1 (pPBIO2728_VIM-1). The genomes from this study (three inner circles) and sequences from a public genome (*E. coli* E-124-4, NCBI accession NZ_PDDP00000000.1) and two plasmids (*E. coli* ECONIH1 plasmid pKPC-629, NCBI accession CP009862.1; *E. coli* R178 plasmid pRH-R178, NCBI accession HG530658.1) (three outer rings) were compared by BLAST (-task megablast -evalue 1e-10 -dust no). Concentric rings are colored by species. The outermost ring shows coding sequences (CDS; colored according to legend) with various common IncN sequence features (e.g., CUP [Conserved Upstream]-controlled genes [*ccg*] [29]) labelled by gene name. The asterisks denote alleles of the IncN pMLST scheme, which match ST7. Note that all isolates in this study and only *E. coli* E-124-4 and *E. coli* R178 carry the *bla*_VIM-1_ resistance gene. The mercury resistance operon, however, was present in all analyzed sequences.

When we aligned all genomes of this study to plasmid 1 (pPBIO2728_VIM-1) of *E. coli* PBIO2728, we identified high similarities of all sequences except PBIO2730 (Figure 1 and Figure S1). 

The breadth of coverage for BLAST hits with at least 99% identity to plasmid 1 ranged from 99.7% (PBIO2726) over 98.2% (PBIO2729) to only 42.3% (PBIO2730), suggesting that three of four isolates (belonging to three different species) carried an almost identical plasmid (IncN ST7) (Figure 1). PBIO2728 (*E. coli*) and PBIO2729 (*K. oxytoca*) originated from the same patient (patient 2) and were both isolated from a rectal swab (screening) on the same day. It thus seems likely that inter-bacterial-species transfer of the VIM-resistance plasmid occurred within the patient (Table 1). We have previously, in a different study, demonstrated that resistance plasmid transmission among Enterobacteriaceae in the clinical setting is common—either within individual or between different patients [26]. Interestingly, PBIO2726 (*C. freundii*) also showed high similarities with the reference plasmid, again suggesting potential dissemination (Figure 1). Note that PBIO2726 was isolated almost four weeks prior to the *E. coli* isolate from an unrelated patient with no epidemiological association (Table 1), possibly indicating that the VIM-resistance plasmid (IncN) circulates in this area. 

**Figure 2:**
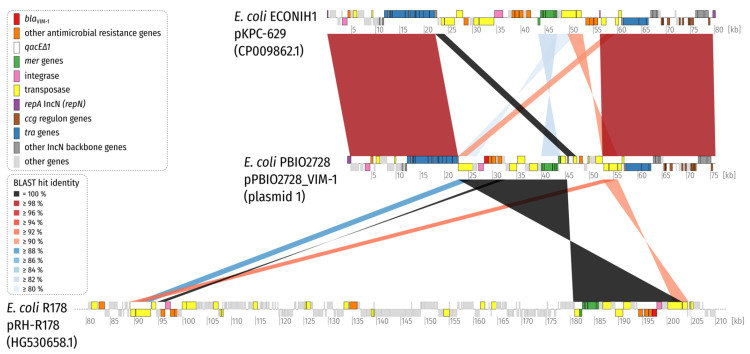
Synteny plot of plasmid 1 (pPBIO2728_VIM-1) of *E. coli* PBIO2728 (ST10) and plasmids from the NCBI nucleotide database. Note that plasmid pRH-R178 (size = 223,382 bp; NCBI accession HG530658.1) of *Escherichia coli* R178 is only displayed partially. For clarity, only BLAST hits with a length of at least 2% of the length of the shorter replicon in the comparison are shown (1520 bp for both comparisons). Coding sequences (CDS) are colored according to the legend. A 22,480 bp region with 100% identity is shared between the plasmids of strains PBIO2728 and R178 and resembles a transposon carrying a class 1 integron (see text and Figure 1).

The VIM-resistance plasmid also showed sequence similarity with public genomes/plasmids that we obtained through a BLAST search against the NCBI nucleotide database (Table S2), whereby no single plasmid showed an overall shared synteny. Instead, the graphical summary of BLAST hits (Table S2) and synteny plotting (Figure 2) suggested a composite nature of the IncN resistance plasmid. While the plasmid of one *E. coli* strain (ECONIH1) shared approximately 67.5% of the plasmid backbone (with at least 99% identity), the plasmid of another *E. coli* strain (R178) shared around 32.9% (Figure 1 and Figure 2). In fact, a large region (22,480 bp) of plasmid pRH-R178 containing the *bla*_VIM-1_ resistance gene as well as the mercury resistance operon is 100% identical to the VIM-1-containing plasmid of PBIO2728. This region harbors an In*110* class 1 integron as described for pRH-R178 (IncHI2) of *E. coli* R178, which was isolated from a livestock farm in Germany in May 2011 [30]. 

All included sequences (the four from this study and from the three external strains) were positive for the mercury resistance operon *mer* (Figure 1 and Figure 2). A study from 2012 suggests that initial mercury resistance first evolved among thermophilic bacteria and that an increase in the complexity of the *mer* operon through continual gene acquisition and evolution led to improved efficiency in the context of a detoxification system [31]. *Mer* operons can therefore be found in both clinical and environmental strains [32]. Interestingly, mercury resistance transposons, e.g., Tn*21*, are often involved in the worldwide distribution of antibiotic resistance determinants [33]. All four isolates showed phenotypically higher mercury MICs (5 µg/mL) compared to a control strain (0.625 µg/mL). Whether and how this system might impact the isolates analyzed in this study remains speculative and will be the focus in prospective investigations.

We compared the *E. coli* ST10 isolate PBIO2728 to a genome of the same sequence type (*E. coli* E-124-4, originally isolated from a Venus clam), which also carried the *bla*_VIM-1_ gene on a plasmid [2]. Our genomic analysis suggests that the two strains were not closely related (20019 SNPs) but shared approximately 35.5% (≥99% identity) of their VIM-plasmids (with pPBIO2728_VIM-1 as reference). Additionally, note that the external ST10 strain carried the *mer* operon as well (Figure 1 and Figure 2).

## 4. Conclusions

Our results demonstrate the circulation of a nearly identical IncN-type VIM-resistance plasmid with a transposon carrying a class 1 integron in three of four bacterial isolates that belong to Enterobacteriaceae in north-eastern Germany.

## Figures and Tables

**Table 1:** Overview of all four investigated isolates and associated geno- and phenotypic data. Only the most important resistance and plasmid incompatibility genes are displayed. Comprehensive results can be found in Table S1. Minimal inhibitory concentrations against the tested antibiotics are displayed in mg/L.

Strain	Species	ST	Patient	Source	Date	Lab	MDR	Antimicrobial Resistance Phenotype (mg/L)							Antimicrobial Resistance Genotype	Incompatibility Types
								CAZ	CIP	GEN	IPM	MEM	TZP	SXT		
PBIO2726	*C. freundii*	396	1	rectal swab	21 June 2020	MVZ	yes	>64 (R)	4 (R)	4 (R)	>16 (R)	>16 (R)	>128 (R)	>320 (R)	*aac(6’)-Ib-G*, *aadA1*, *bla*_CMY-78_, *bla*_VIM-1_, *dfrA14*, *fosA7.2*, *qnrS1*, *sul1*, *sul2*, *tet*(34)	IncN (ST7), IncFII(S), IncFII(SARC14)
PBIO2728	*E. coli*	10	2	rectal swab	26 July 2020	MVZ	yes	>64 (R)	0.5 (I)	4 (R)	>16 (R)	>16 (R)	>128 (R)	>320 (R)	*aac(6’)-Ib-G*, *aadA1*, *bla*_VIM-1_, *dfrA14*, *qnrS1*, *sul1*, *tet*(34)	IncN (ST7), IncFIA(HI1), IncFIB(K), IncFII(p96A), IncFII
PBIO2729	*K. oxytoca*	168	2	rectal swab	26 July 2020	MVZ	yes	>64 (R)	2 (R)	8 (R)	>16 (R)	>16 (R)	>128 (R)	>320 (R)	*aac(6’)-Ib-G*, *aadA1*, *bla*_OXY-6-1_, *bla*_VIM-1_, *dfrA14*, *fosA*_gen, *oqxA10*, *oqxB20*, *qnrS1*, *sul1*, *tet*(34)	IncN (ST7), IncFIB(K)
PBIO2730	*K. oxytoca*	172	3	sternal wound	5 August 2020	IMD	yes	>64 (R)	4 (R)	2 (R)	>16 (R)	>16 (R)	>128 (R)	>320 (R)	*aac(6’)-Ib-G*, *aph(3’’)-Ib*, *aph(6)-Id, bla_OXA-10_, bla_OXY-6-2_*, *bla*_VIM-1_, *catA1*, *cmlA5*, *dfrA14*, *fosA*_gen, *mcr-9.1*, *oqxA10*, *oqxB20*, *qnrS1*, *sul1*, *tet*(34)	IncHI2, IncHI2A, IncFII(pCRY)

ST: sequence type. CAZ: ceftazidime. CIP: ciprofloxacin. GEN: gentamicin. IPM: imipenem. MEM: meropenem. TZP: piperacillin-tazobactam. SXT: trimethoprim-sulfamethoxazole. MDR: multidrug-resistant. Lab: Laboratory. R: resistant. I: intermediate. MVZ: Medizinisches Versorgungszentrum. IMD: Institut für Medizinische Diagnostik. Inc: incompatibility.

## Data Availability

The sequence data for this study has been deposited in the European Nucleotide Archive (ENA) at EMBL-EBI under accession number PRJEB44350 (https://www.ebi.ac.uk/ena/browser/view/PRJEB44350; accessed on 15 April 2021).

## Supplementary Materials

The following are available online at https://www.mdpi.com/article/10.3390/microorganisms9071345/s1, Table S1: Overview of all detected genes compared to the respective databases. Values indicate percent (%) coverage and identity (the latter in parentheses). Table S2: BLAST search results for *E. coli* PBIO2728 plasmid pPBIO2728_VIM-1 against the NCBI nucleotide database sorted by query coverage (descending). Highlighted entries are included in the analyses of this study. Figure S1: Plasmid profile analysis of all investigated isolates. Strain names are colored according to species as in Figure 1.
