# Supplementary material for: Nearly Identical Plasmids Encoding VIM-1 and Mercury Resistance in Enterobacteriaceae from North-Eastern Germany
Source: Microorganisms. 2021 Jun 22;9(7):1345. doi: 10.3390/microorganisms9071345 (PMC8305640; doi:10.3390/microorganisms9071345)
Supplement: Supplementary file 1 [file microorganisms-09-01345-s001.zip › microorganisms-1214325-supplementary/microorganisms-1214325-S/microorganisms-1214325-S-F.pdf]

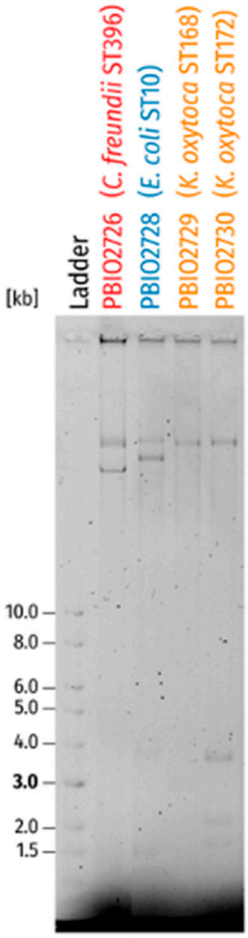

Figure S1: Plasmid profile analysis of all investigated isolates. Strain names are colored according to species as in Figure 1.
